# Quorum Sensing Activity and Hyphal Growth by External Stimuli in the Entomopathogenic Fungus *Ophiocordyceps sinensis*

**DOI:** 10.3390/insects11040205

**Published:** 2020-03-26

**Authors:** Guiqing Liu, Li Cao, Xuehong Qiu, Richou Han

**Affiliations:** Guangdong Key Laboratory of Animal Conservation and Resource Utilization, Guangdong Public Laboratory of Wild Animal Conservation and Utilization, Guangdong Institute of Applied Biological Resources, Guangzhou 510260, China; pepsiliu81@163.com (G.L.); caol@giabr.gd.cn (L.C.); xhqiu@126.com (X.Q.)

**Keywords:** *Ophiocordyceps sinensis*, fungal dimorphism, inoculum density, nutrient, quorum sensing molecule, fungal metabolite, insect hormone

## Abstract

The entomopathogenic fungus *Ophiocordyceps sinensis* is one of the best known and most precious medicines and health food in China. The blastospores-hyphae (dimorphism) transition of this fungus in host hemolymph is critical for the virulence and the mummification of host larvae. To regulate this transition, the effects of inoculum density and fifteen chemicals including fungal nutrients, fungal metabolites, quorum-sensing molecules (QSMs) and insect hormones on the dimorphism in *O. sinensis* were investigated in vitro. The blastospores tended to exhibit budding growth when inoculated at 10^7^ blastospores per mL, and hyphal growth at concentrations lower than 10^6^ blastospores per mL. At 10^5^ blastospores per mL, the percentage of hyphal formation decreased with the addition of filtered spent medium containing 10^7^ blastospores per mL, indicating the quorum-sensing effect. Blastospores-hyphae transition in this fungus by fifteen chemicals was varied from no response to dimorphic reversion. The addition of *N*-acetylglucosamine at three concentrations significantly stimulated hyphal formation while inhibiting budding growth. For the first time, insect hormone 20-hydroxyecdysone was found to be involved in the hyphal formation in fungi. These results open new possibilities to regulate the dimorphism, which would be beneficial for the cultivation of the Chinese cordyceps.

## 1. Introduction

*Ophiocordyceps sinensis* is a rare entomopathogenic fungus that parasitizes the larva of the ghost moth *Thitarodes* spp. and then the stroma grows from the head of the mummified larva. This mysterious insect-fungus parasitic complex, also called the Chinese cordyceps [1], has been used for medicinal treatment and health food since the 15th century [2,3,4,5]. The Chinese cordyceps are endemic to the Tibetan plateau at altitudes between 3000 and 5200 m and its natural yield is very limited [6,7]. In the last decade, artificial cultivation of *O. sinensis* fruiting bodies on rice media and the host caterpillar *Thitarodes* sp. has been established [8,9,10]. However, the low mummification rate post-infection is still an unresolved obstacle for commercial cultivation of the Chinese cordyceps [11,12,13]. *O. sinensis* could colonize the larva’s hemocoel for more than one year without killing the host larva if the blastospores do not turn into a differentiation phase to form invasive hyphae [14]. Compared with other entomopathogenic fungi, such as *Metarhiziurn anisopliae* and *Beauveria bassiana*, which cause the death of host larvae within a few days [15,16,17], *O. sinensis* took a long time to kill its host larvae.

**Figure 1:**
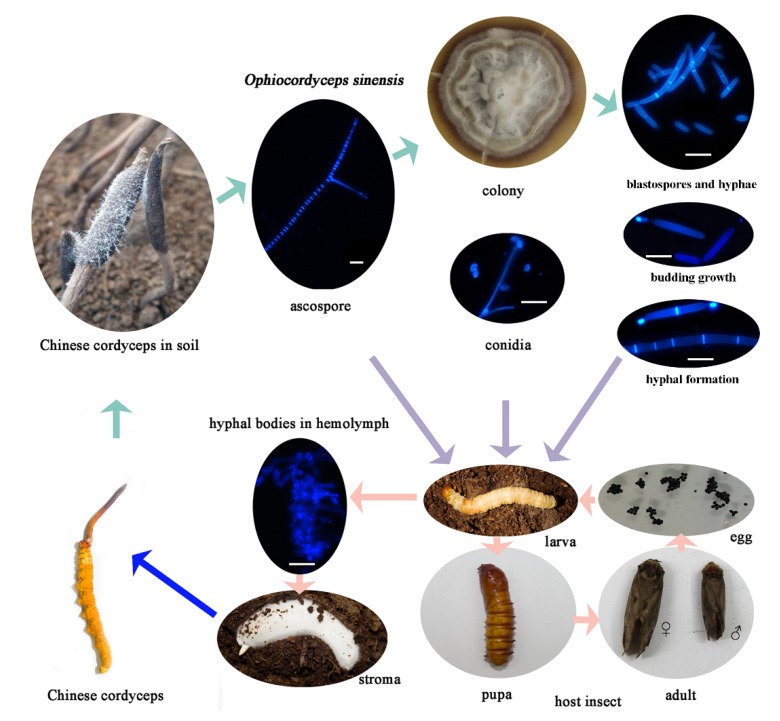
The life cycle of *Ophiocordyceps sinensis* and the development process of the Chinese cordyceps. The bar indicates 20 μm.

Like species within the genera *Beauveria*, *Metarhizium,* and *Isaria* [18], *O. sinensis* also exhibits an in vivo dimorphic developmental process [14]. The blastospores grow exponentially by budding growth in the hemocoel without apparent damage to the host larva. Blastospores were found to have the ability to evade recognition by the host hemocytes in *M. anisopliae* [19]. As previously reported, the ability of pathogenic fungi to switch between yeast cells and hyphae is crucial for the pathogenicity both in the plant pathogen *Ustilago maydis* and in the human prevalent pathogen *Candida albicans* [20,21,22,23,24]. In the entomopathogen *Metarhizium* (*Nomuraea*) *rileyi*, the hyphal tips are coated with a layer of laminin-binding material, which allows hypha to attach to the basement membrane that encases the host tissues [25]. *O. sinensis* is a polymorphic fungus with a complex life cycle (Figure 1), the trigger(s) responsible for the switch from the proliferation of hyphal bodies by budding growth to the apical growth program in these entomopathogenic fungi is unknown. So, this study on the dimorphism in *O. sinensis* is necessary for understanding the mummification process and for developing effective methods to regulate this dimorphism during the artificial cultivation of Chinese cordyceps.

In other fungal systems, a number of external inputs such as nutrients, temperature, pH, CO_2_ and chemicals and quorum sensing molecules (QSMs) for the morphological transition in dimorphic fungi have been identified [26,27,28,29]. At a threshold cell population density, also described as quorum density, the secreted inducers regulate either the change of cell behavior or the transition of cell phenotype [30]. To date, nitrogen sources such as L-proline and *N*-acetyl-glucosamine and several QSMs such as tryptophol, farnesol, tyrosol, farnesoic acid are found to function in *C. albicans* [24,30,31]. The quorum sensing behavior maybe host- and strain-specific [31,32,33,34,35]. Under in vitro culture conditions, biological active ecdysteroid-22-oxidase, an enzyme inactivating the insect hormone ecdysone to prevent insect metamorphosis, was identified at the hyphal culture stage [36]. In addition, the fungal metabolite ergosterol peroxide has been found to inhibit melanization [37,38]. In *C. albicans*, the yeast-hypha transition could be controlled by small organic chemicals, for example, doxycycline, BH3I-1, and phorbasin H which provides new possibilities for the development of antifungal agents [28,39,40].

Quorum sensing (QS) activity in *Ophiocordyceps* fungi has not been described before. In the following investigation, the inoculum effects on the blastospores-to-hyphae transition of *O. sinensis* were determined, and the evidence for quorum-sensing was provided by an in vitro assay to monitor the cell phenotype. Furthermore, the effects of fifteen chemicals, including nutrient input, known fungal QSMs, fungal metabolites, and insect hormones on the dimorphism in *O. sinensis* was determined using an in vitro assay in a dose-response model.

## 2. Materials and Methods

### 2.1. Strain, Culture Media and Culture Conditions

Strain KD1223 (GDMCC 60594) was isolated from fruiting bodies in Sichuan, China and maintained at −80 °C in 40% glycerol in Guangdong Institute of Applied Biological Resources, Guangzhou, China. The stock culture was transferred to liquid potato glucose medium supplemented with 10% peptone (PPD, pH 6.2) and shaken for 40 days at 13 °C on a 100 rpm rotary shaker [41]. The resulting fungal cultures were inoculated and grown for 40 days in liquid PM medium (maltose instead of dextrose in PPD, pH 6.3) at 13 °C on a 100 rpm rotary shaker. Blastospores were harvested by using three layers of lens papers to remove hypha and large particles, pelleted from the filtrate by centrifugation at 5000 rpm for 10 min at 10 °C, washed twice and resuspended in 5 mL sterile 1 × phosphate buffered saline (PBS, pH 7.2) solution, and counted using a hemocytometer and a light microscope (Eclipse 80i; Nikon, Japan) at 400×. The blastospores were used immediately or stored at 4 °C not more than two days before being used as the inocula.

### 2.2. Chemicals for Bioassay

Fifteen chemicals were used to determine their effects on blastospores-hyphae dimorphism: three nutrient substances including L-proline (Sigma-Aldrich, St. Louis, MO, USA), *N*-acetyl-d-glucosamine (Sigma-Aldrich), and β-1,3-glucan from *Euglena gracilis* (Sigma-Aldrich); three fungal metabolites including ergosterol (Sigma-Aldrich), ergosterol peroxide (Carbosynth, Berkshire, UK), and zaragozic acid A (Sigma-Aldrich); three quorum-sensing molecules identified in *C. albicans*, including trans-farnesol (Sigma-Aldrich), tyrosol (Sigma-Aldrich), and β-phenylethanol (Sigma-Aldrich, MO, USA); six insect hormones and their analogs including juvenile hormone I and II (JH I and II, TRC, Toronto, ON, Canada), juvenile hormone III (JHIII, Sigma-Aldrich), methyl farnesoate (Echelon Biosciences, Salt Lake City, UT, USA), methoprene (TRC, Toronto, ON, Canada) and (+)-20-hydroxyecdysone (TRC, Toronto, ON, Canada). Trans-farnesol was prepared in dimethyl sulphoxide (DMSO, Sigma-Aldrich) to a final concentration of 2 mM, 0.2 mM, 0.02 mM, and 0.005 mM. Tyrosol and β-phenylethanol were prepared in sterile distilled water to a final concentration of 2 mM, 1 mM, 0.2 mM, and 0.02 mM. Ergosterol was prepared in boiling ethanol (EtOH) to a final concentration of 2 mM, 1 mM, 0.2 mM, and 0.02 mM. Both Methoprene and (+)-20-hydroxyecdysone were prepared in DMSO to a final concentration of 2 mM, 1 mM, 0.2 mM, and 0.02 mM. Another nine chemicals were also prepared in DMSO, such as *N*-acetyl-glucosamine and L-proline at 2 mM, 0.2 mM, and 0.02 mM solutions and β-1,3-Glucan at 1 mg mL^−1^, 0.1 mg mL^−1^ and 0.05 mg mL^−1^, JH I, JH II, ergosterol peroxide and zarogozic acid A at 0.5 mM, 0.05 mM, and 0.01 mM, JHIII and methyl farnesoate as 1 mM, 0.5 mM, and 0.05 mM, respectively. All the test concentrations were set by referring to the previous studies [42,43,44]. The wells with or without DMSO and EtOH were set up to assess any effects of DMSO and EtOH on blastospores-to-hyphae transition (Figure S1). The final concentration of the solvent DMSO and EtOH was 0.1% and 3.6%, respectively.

### 2.3. Effects of Fungal Inoculum Density on the Blastospores-to-Hyphae Transition

Liquid shake cultures were made in 250 mL flasks containing 50 mL of PM or PMG (PM with the addition of 0.5% milled fresh greater wax moth) media. Then, blastospores were inoculated into PM and PMG media to a final concentration of 1 × 10^7^, 1 × 10^6^ and 1 × 10^5^ blastospores per mL. Inoculated flasks were incubated at 13 °C on a 100 rpm rotary shaker. All treatments were conducted in triplicate. Fungal cultures were collected from the flasks every two days, equally mixed with Calcofluor White (Sigma-Aldrich), and the blastospores and hyphae were counted. The morphology of the fungal cultures was monitored 8 days after inoculation.

To verify the possible extracellular molecules present in the fungal cultures for the blastospores-to-hyphae transition, the spent media generated by inoculating 50 mL of PM medium in 250 mL flasks with a final concentration of 10^7^ blastospores per mL and incubating at 13 °C on a 100 rpm rotary shaker for 8 days were centrifuged at 5000 rpm at 4 °C for 10 min and the supernatants were filtered with 0.45 µm filters (Pall Corporation, Puerto Rico, USA). The resulting supernatant was used to prepare a fresh PM medium, in which the final volume of the supernatant accounted for 80% and 50%. Then, blastospores were inoculated into the flask (50 mL fresh PM medium in 250 mL flask) with a final concentration of 10^5^ blastospores per mL and incubated at 13 °C on a 100 rpm rotary shaker. The fungal phenotype was observed under microscopy at 6 and 8 days at 13 °C after inoculation. Ten µL aliquot culture solutions from each well were sampled and immediately mixed with 10 µL aliquot of Calcofluor White. A fluorescence microscope (IX73; Olympus, Tokyo, Japan) was used to examine the blastospores and hyphae at 400×. Numbers of fungal forms (blastopores, budding blastospores, and hyphae) were counted. Blastospores with buds were counted as budding blastospores if they had a visible constriction at the bud site, and blastospores with single or multiple septa were classified as hyphae. The samples from the flasks with inocula containing less than 10^6^ blastospores per mL were concentrated five fold by 10 min of centrifugation at 5000 rpm before microscopic examination. At least 200 fungal forms (blastospores, budding blastospores or hyphae) were counted for each sample.

### 2.4. Effects of Chemicals on the Blastospores-to-Hyphae Transition

Bioassays to evaluate the fifteen chemicals for blastospores-to-hyphae transition were carried out in a dose-response model, in sterile 96-well microtiter plates (Corning Incorporated, Oneonta, NY, USA) that contained 180 µL PM medium in each well. An aliquot of 10 µL of the chemical solution was pipetted into PM medium to the set final concentration above and mixed with a microporous quick shaker (Kylin-Bell, Haimen, China). Then, an aliquot of 10 µL blastospores was added to the well to a final concentration of 1 × 10^7^ or 1 × 10^5^ blastospores per mL. 0.1% DMSO and 3.6% EtOH were added to wells as controls, respectively. The phenotype of blastospores was observed under microscopy daily after the inoculation. Fungal cultures were sampled at day 8 after inoculation (before new blastospores formed by budding growth shedding from their parent blastospores) and equally mixed with Calcofluor White and counted. Three well replicates were established for each treatment.

The accumulation of fungal biomass was evaluated in the following three media. The fungal strain KD1223 was cultured on PM medium (liquid PM medium supplemented with 1.5% agar) for 60 days at 13 °C. The colonies with a diameter of 0.9 mm were transferred to 250 mL flasks containing 150 mL liquid PM (pH 6.39), PMP (PM with the addition of 0.1% L-proline, pH 6.45) or PMN (PM with the addition of 0.1% *N*-acetyl-d-glucosamine, pH 6.39). Flasks were incubated on a 100 rpm rotary shaker at 13 °C. An aliquot of 1 mL fungal cultures incubated after 15, 30, and 45 days were sampled, respectively from each flask for blastospores counting and observation of microcycle conidiation in a hemocytometer. The fungal biomass from each flask after 60 days were harvested with three layers of lens papers and transferred to a 50 mL centrifuge tube. The filtered hyphae were pre-weighted and freeze-dried to a constant weight using Savant freeze drier (Thermo Fisher Scientific, Waltham, MA, USA). The average dry weight of mycelium from six flasks was calculated.

### 2.5. Data Analysis

The percentages of blastospores, budding blastospores and hyphae are a total of 100%, but only the percentages of budding blastospores or hyphae are presented. The percentage of budding blastospores was calculated by the ratio of the numbers of budding blastospores to the total numbers of all fungal forms. The percentage of hyphal formation was calculated by the ratio of the numbers of blastospores exhibiting hyphal growth to the total numbers of all fungal forms.

PROC Nonparametric Tests with the command ‘Sample K-S’ were used for the analysis of the normal distribution of data. PROC GLM with the command ‘univariate’ was used for the analysis of the interaction of concentrations of the chemicals and inoculum density (SPSS17.0, SPSS Inc., Chicago, IL, USA). Significant interaction with concentrations of the chemicals and inoculum density was observed. Therefore, data were analyzed by one-way ANOVA, using Tukey’s honestly significant difference test (*p* < 0.05) or *t*-tests for experiments with two treatments.

## 3. Results

### 3.1. Effect of Inoculum Density on Fungal Forms and QS Activity

**Figure 2:**
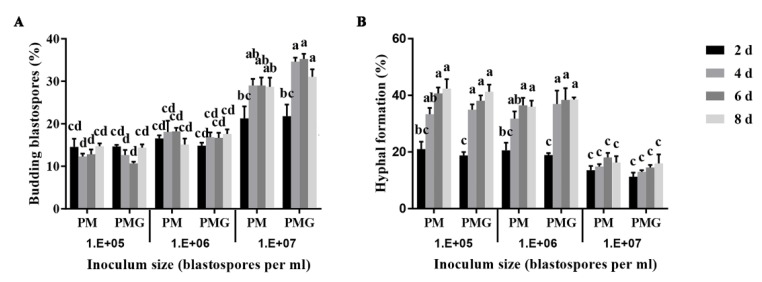
Effects of inoculum density on blastospores budding growth (**A**) and hyphal formation (**B**) of *Ophiocordyceps sinensis* in liquid PM and PMG culture media. Different letters indicated the significant levels at *p* < 0.05.

Blastospores exhibited budding growth and hyphal formation simultaneously in the liquid culture medium (Figure 1). Different fungal forms and their role in the fungal pathogenicity are shown in Figure 1. The inoculum density significantly affected blastospores-to-hyphae transition (*p* < 0.001). The effect of inoculum density on blastospores budding growth and hyphal formation showed no significant difference in PM and PMG media. Blastospores were found more likely to develop into budding blastospores when inoculated at a final density of 10^7^ blastospores per mL, and into hyphae at 10^6^ and 10^5^ blastospores per mL both in PM and PMG medium (Figure 2).

**Figure 3:**
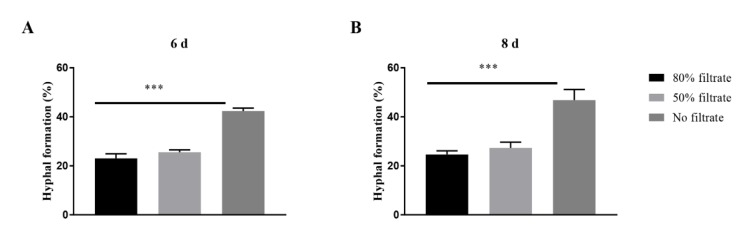
Effects of the spent medium on the hyphal formation in *Ophiocordyceps sinensis* when 10^5^ blastospores per mL were inoculated in liquid PM medium after 6 days (**A**) and 8 days (**B**). *p* < 0.001 (***).

To investigate whether extracellular molecules with QS activity were produced, supernatants of the spent media containing 10^7^ blastopores per mL were used to prepare fresh PM medium containing 10^5^ blastospores per mL, to evaluate its effect on hyphal formation. The addition of 50% and 80% (vol/vol) supernatants caused a significant decrease in the percentages of hyphal formation incubated both 6 days and 8 days after inoculation (Figure 3).

### 3.2. Blastospores-Hyphae Transition of O. sinensis to External Stimuli

**Figure 4:**
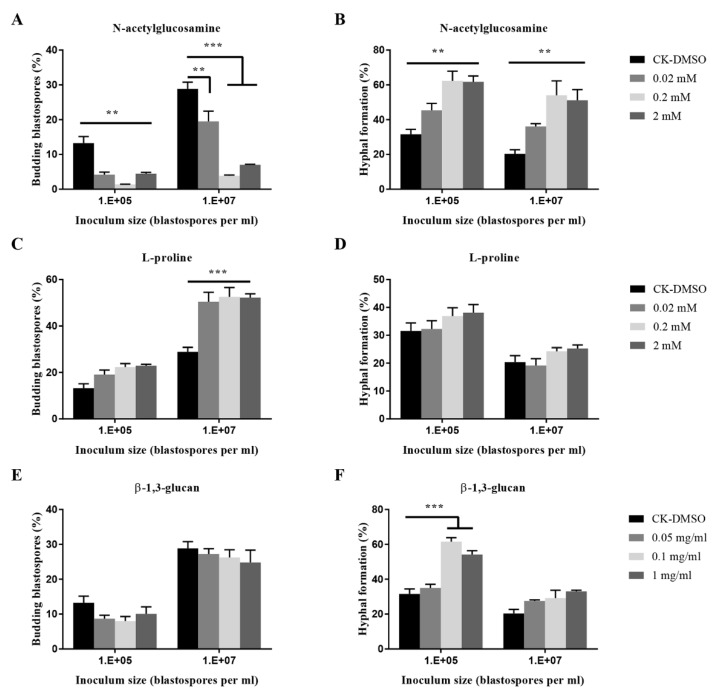
Effects of nutrient inputs on blastospores budding growth (**A**,**C**,**E**) and hyphal formation (**B**,**D**,**F**) of *O. sinensis* in liquid PM medium 8 days after inoculation. *p* < 0.01 (**) and *p* < 0.001 (***).

As shown in Figure 4, fungal cultures treated with L-proline exhibited a significant increase of blastospore budding growth when inoculated at 10^7^ blastospores per mL. It did not affect the hyphal formation. At inocula of 10^7^ and 10^5^ blastospores per mL, *N*-acetyl-glucosamine significantly promoted hyphal formation but inhibited budding growth at three concentrations. When treated with β-1,3-glucan, a fungal cell wall polysaccharide, fungal cultures exhibited chemical dose-dependent promotion of hyphal formation when inoculated at 10^5^ blastospores per mL. β-1,3-glucan at mg mL^−1^ and 0.1 mg mL^−1^ significantly stimulated the hyphal formation when 10^5^ blastospores per mL were inoculated.

**Figure 5:**
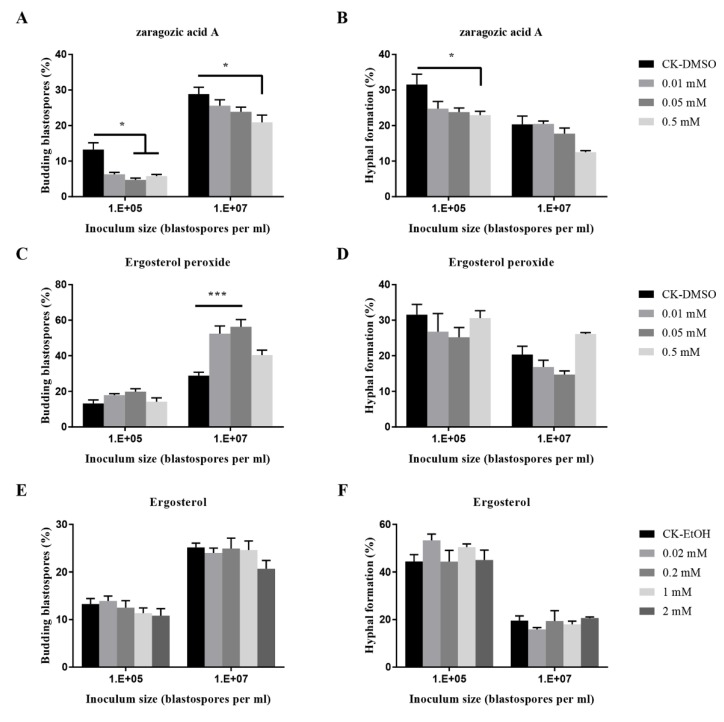
Effects of fungal metabolites on budding growth (**A**,**C**,**E**) and hyphal formation (**B**,**D**,**F**) of *O. sinensis* in liquid PM medium 8 days after inoculation. *p* < 0.05 (*) and *p* < 0.001 (***).

The blastospores-hyphae transition of *O. sinensis* by fungal metabolites such as zaragozic acid A, ergosterol peroxide, and ergosteral was varied (Figure 5). Zaragozic acid A significantly inhibited the budding growth at an inoculum of 10^7^ blastospores per mL, and hyphal formation at 10^5^ blastospores per mL; Ergosterol peroxide at 0.01 mM and 0.05 mM significantly promoted the budding growth when inoculated at 10^7^ blastospores per mL.

**Figure 6:**
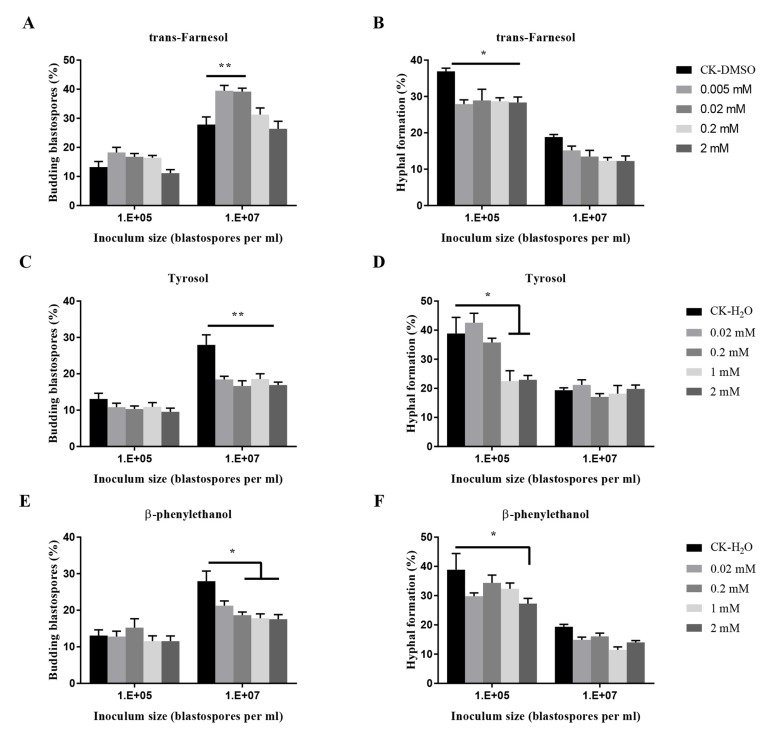
Effects of quorum sensing molecules (QSMs) on budding growth (**A**,**C**,**E**) and hyphal formation (**B**,**D**,**F**) of *O. sinensis* in liquid PM medium 8 days after inoculation. *p* < 0.05 (*) and *p* < 0.01 (**).

The effects of three known quorum-sensing molecules from other fungi on the transition of *O. sinensis* were dose-dependent (*p* < 0.001) (Figure 6). Trans-farnesol at 0.02 mM and 0.005 mM stimulated significant budding growth of blastospores at 10^7^ blastospores per mL, and at four tested concentrations, significantly inhibited hyphal formation at 10^5^ blastospores per mL. Tyrosol at four tested concentrations exhibited significant inhibition of blastospores budding growth when inoculated at 10^7^ blastospores per mL, and at 1 mM and 2 mM, significant inhibition of hyphal formation at 10^5^ blastospores per mL. β-phenylethanol at 2 mM significantly inhibited hyphal formation.

**Figure 7:**
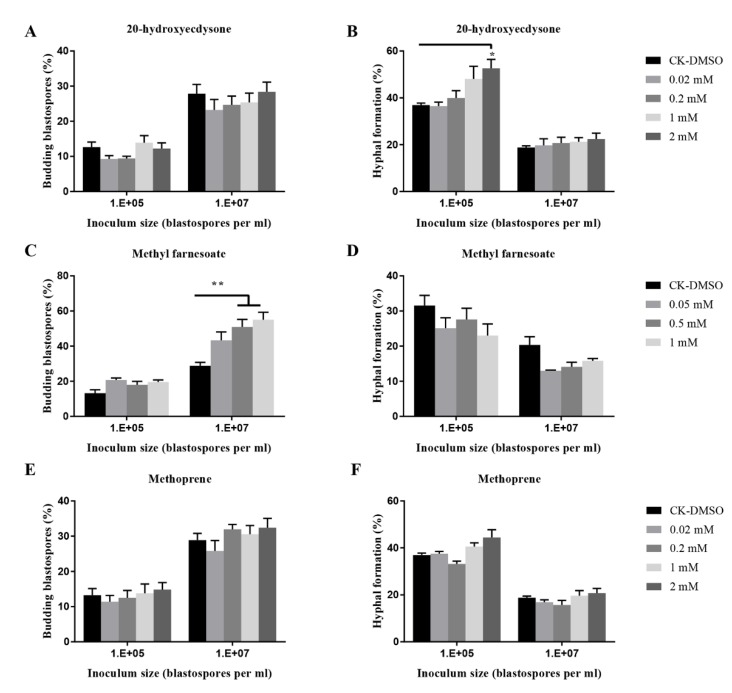
Effects of insect hormones and their analogs on budding growth (**A**,**C**,**E**) and hyphal formation (**B**,**D**,**F**) of *O. sinensis* in liquid PM medium 8 days after inoculation. *p* < 0.05 (*) and *p* < 0.01 (**).

In addition, compared to the control, methyl farnesoate at 2 mM significantly stimulated blastospore budding growth when 10^7^ blastospores per mL were inoculated; 20-hydroxyecdysone at 2 mM significantly promoted hyphal growth when 10^5^ blastospores per mL were inoculated (Figure 7), whereas the other four insect hormones (JHI, II, III, and methoprene) did not stimulate or inhibit hyphal formation (Figure 7 and Figure S2).

### 3.3. Effects of Proline and N-acetyl-glucosamine on Biomass Accumulation of O. sinensis

The blastospore production and total biomass accumulation in liquid shake cultures of *O. sinensis* were strongly influenced by the nitrogen sources (Table 1). The addition of proline to PM medium stimulated the production of blastospores and the accumulation of total biomass, whereas low numbers of blastospores and total biomass were obtained with the addition of *N*-acetyl-glucosamine. The conidial production by microcycle conidiation was influenced neither by proline nor by *N*-acetyl-glucosamine.

## 4. Discussion

Blastospores-hyphae transition is related to fungal pathogenicity and virulence to mammals, plants, and insects [23], and the connection between the blastospores-hyphae transition and the virulence of *O. sinensis* fungus to host insect larvae is also observed [14]. It is necessary to explore the factors involved in blastospores-hyphae dimorphism, to regulate the mummification process during the artificial cultivation of the Chinese cordyceps.

In this study, high blastospores density promoted their proliferation by budding growth and the addition of the spent medium containing 10^7^ blastospores per mL into the fungal cultures with 10^5^ blastospores per mL did cause a significant decrease of hyphal formation, indicating the presence of QSMs. To the best of our knowledge, this is the first report of quorum-sensing activity in *O. sinensis* fungus. Quorum-sensing has been found in yeast such as *C. albicans*, *Saccharomyces cerevisiae*, *Yarrowia lipolytica,* and several fungi such as *Ophiostoma (=Ceratocystis) ulmi*, *Tremella fuciformis* and *U. maydis*, and the QSM was specific to different species [26,45,46,47,48,49,50,51].

For *C. albicans*, farnesol secreted at high inoculum cell density acts as the QSM to promote fungal budding growth and to inhibit hyphal formation [45]. Tyrosol, another QSM, promotes hyphal growth of *C. albicans* [50]. However, the QSM for *O. ulmi* was distinct from farnesol as farnesol had no effect on dimorphism in *O. ulmi* [51]. Oxylipins were considered to be involved in the control of yeast-to-hypha transition in *O. ulmi* by in vitro test [35]. Other molecules such as cyclic sesquiterpenes were also detected and considered to be responsible for the budding growth of *O. floccosum* [52]. The addition of exogenous farnesol up to 1 mM promoted the growth of hyphae in *Penicillium decumbens* [52]. In the present study, farnesol was found to stimulate blastospores budding growth at concentrations of 0.005 mM and 0.02 mM and inhibited hyphal formation at concentrations ranging from 0.005 mM to 2 mM when 10^5^ blastospores per mL were inoculated. Tyrosol was found to inhibit both budding growth and hyphal formation at 2 mM and 1 mM. β-phenylethanol, a quorum-sensing molecule from *S. cerevisiae* with enhancing activity for pseudohyphal growth at a relatively low concentration, had no effect in *C. albicans* [33,48]. In this study, β-phenylethanol at concentrations higher than 0.02 mM exhibited inhibition of budding growth at 10^7^ blastospores per mL, and at 2 mM strongly inhibited hyphal formation when 10^5^ blastospores per mL were inoculated. These results indicated that exogenous QSMs from other fungi could function on the dimorphism in *O. sinensis* fungus.

*N*-acetylglucosamine is the monomer of the polysaccharide chitin, an essential structural component of the fungal cell wall and the arthropod exoskeleton. It was found to be a potent inducer of the transition from yeast to hyphal form in *C. albicans*, *Histoplasma capsulatum,* and *Blastomyces dermatitidis* [53], but an inhibitor of hyphal growth in *Neurospora crassa* [54]. In this study, *N*-acetylglucosamine strongly increased hyphal formation and inhibited blastospores budding growth both at 10^7^ and 10^5^ blastospores per mL, whereas L-proline significantly promoted blastospores budding growth when 10^7^ blastospores per mL were inoculated. It seems that *O. sinensis* responds to *N*-acetylglucosamine in a similar way in *C. albicans*.

Proline was also found to induce the yeast morphology when higher than 10^6^ blastospores per mL from *O. ulmi* were inoculated [50]. Blastospores production and total biomass accumulation in liquid shake cultures of *O. sinensis* strongly increased with the addition of L-Proline, but significantly decreased with the addition of *N*-acetylglucosamine. Zaragozic acid A is a powerful antifungal antibiotic, which inhibits the growth and targets steps in sterol biosynthesis in *C. albicans* [55]. Both blastospores budding growth and hyphal formation in *O. sinensis* were inhibited by zaragozic acid A, suggesting that sterol biosynthesis in *O. sinensis* might be blocked.

Interestingly, 20-hydroxyecdysone at 2 mM promoted hyphal formation, but other exogenous insect hormones such as JHI, II, III, and methoprene did not regulate blastospores-to-hyphae transition. Another study also demonstrated that insect hormones including ecdysone, JHI, II, III, methoprene and methyl farnesoate at 0.5 mM did not induce hyphal bodies-to-hyphae transition in *M. rileyi* [43]. This is the first time to discover that insect hormone 20-hydroxyecdysone regulates hyphal formation in fungi. It would be interesting to see whether the fungal response to this insect hormone occurs in vivo insect hemolymph.

## 5. Conclusions

The present study provided evidence for the presence of the quorum-sensing system in *O. sinensis*. QSMs identified in *C. albicans*, such as farnesol and tyrosol, had significant effects on the dimorphism of *O. sinensis*. The blastospores-hyphae transition in this fungus was regulated by some of the external stimuli, such as L-proline and *N*-acetylglucosamine. These results provide useful data to better understand the biology and pathogenicity of *O. sinensis* for the improved cultivation of the Chinese cordyceps.

## Figures and Tables

**Table 1:** Effect of L-proline and *N*-acetylglucosamine on biomass accumulation of *O. sinensis*.

Medium	Spore Yield 15 Days after Inoculum		Spore Yield 30 Days after Inoculum		Spore Yield 45 Days after Inoculum		Biomass (g) 60 Days after Inoculum
	Conidia (×10^8^ per mL)	Blasto-Spores	Conidia (×10^8^ per mL)	Blasto-Spores (×10^5^ per mL)	Conidia (×10^8^ per mL)	Blastospores (×10^5^ per mL)	
PM	1.81 ± 0.35a	0	4.22 ± 0.71a	5.03 ± 0.84b	8.53 ± 0.89a	11.37 ± 1.74b	2.87 ± 0.11b
PMP (+proline)	2.09 ± 0.18a	0	5.03 ± 0.93a	25.81 ± 4.21a	9.49 ± 0.44a	578.13 ± 95.15a	4.08 ± 0.37a
PMN (+GlcNAc)	1.86 ± 0.17a	0	5.17 ± 0.68a	0.23 ± 0.03c	6.00 ± 0.12a	0.32 ± 0.03c	1.44 ± 0.16c

Note: Means (±SE) with different letters indicate a significant difference at *p* < 0.05.

## Supplementary Materials

The following are available online at https://www.mdpi.com/2075-4450/11/4/205/s1, Figure S1: Effect of solvent DMSO (A-B) and EtOH (C-D) on blastospores budding growth and hyphal formation, Figure S2: Effect of insect hormones (JHI, JHII and JHIII) on blastospores budding growth (A, C, E) and hyphal formation (B, D, F).
