# Supplementary material for: Quorum Sensing Activity and Hyphal Growth by External Stimuli in the Entomopathogenic Fungus Ophiocordyceps sinensis
Source: Insects. 2020 Mar 26;11(4):205. doi: 10.3390/insects11040205 (PMC7240566; doi:10.3390/insects11040205)
Supplement: Supplementary file 1 [file insects-11-00205-s001.pdf]

Insects

Supplementary Materials

Quorum Sensing Activity and Hyphal Growth by External Stimuli in the Entomopathogenic Fungus *Ophiocordyceps sinensis*

Guiqing Liu [1], Li Cao [1], Xuehong Qiu [1] and Richou Han [1,*]

Guangdong Key Laboratory of Animal Conservation and Resource Utilization, Guangdong Public Laboratory of Wild Animal Conservation and Utilization, Guangdong Institute of Applied Biological Resources, Guangzhou, China

Corresponding author: Prof. Richou Han, hanrc@giabr.gd.cn

Supplementary Materials

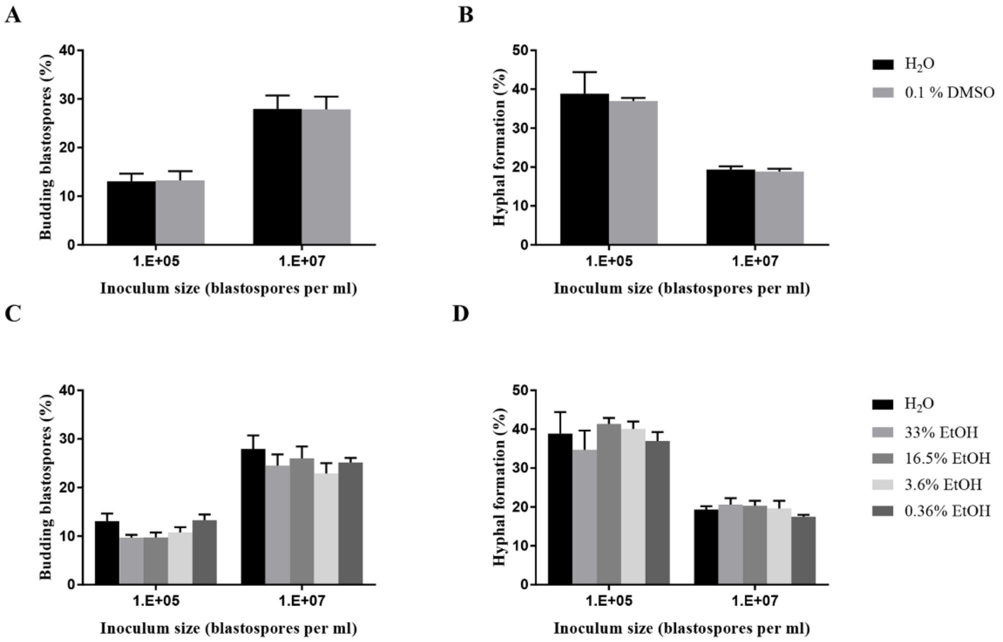

Supplementary Figure S1. Effect of solvent DMSO (A-B) and EtOH (C-D) on blastospores budding growth and hyphal formation.

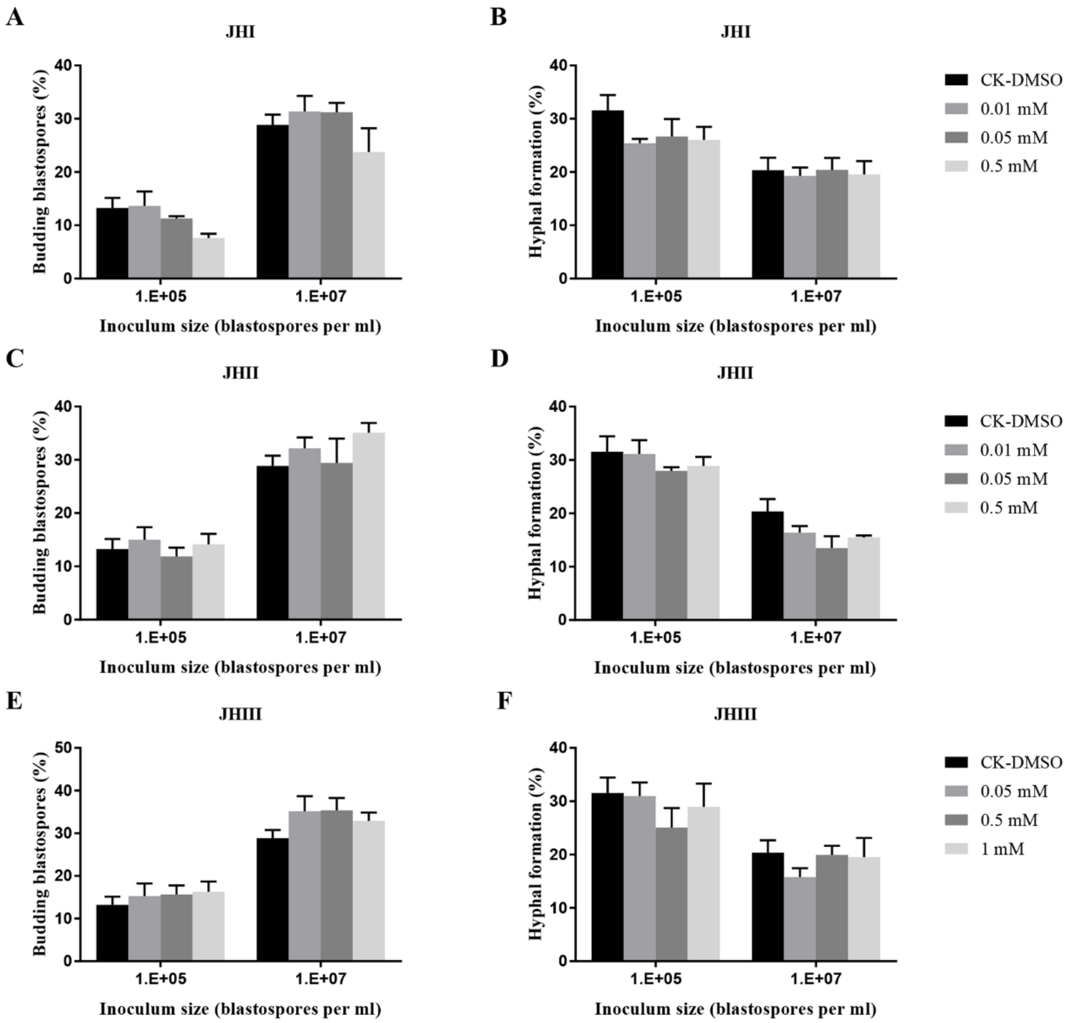

Supplementary Figure S2. Effect of insect hormones (JHI, JHII and JHIII) on blastospores budding growth (A, C, E) and hyphal formation (B, D, F).
